# Experiences of reduced work hours for nurses and assistant nurses at a surgical department: a qualitative study

**DOI:** 10.1186/s12912-017-0210-x

**Published:** 2017-04-04

**Authors:** Kristina Gyllensten, Gunnar Andersson, Helena Muller

**Affiliations:** 1 Department of Occupational and Environmental Medicine, University of Gotenburg and Sahlgrenska University Hospital, Gothenburg, Sweden; 2 Department of Psychology, University of Gotenburg, Gothenburg, Sweden

**Keywords:** Reduced work hours, Nurses, Assistant nurses, Qualitative research, Psychosocial working environment, Work-life balance

## Abstract

**Background:**

There is a shortage of registered nurses in the European Union (EU), and job dissatisfaction and perceived high work–family conflict have been identified as causes of nursing staff turnover. Reducing work hours is an organisational intervention that could have a positive effect on nurses’ and assistant nurses’ job satisfaction, work–life balance, and willingness to stay in the job. An orthopaedic surgery department at a large hospital in Sweden introduced reduced work hours for nurses and assistant nurses in order to improve the working situation. The aim of the study was to investigate the experiences of reduced work hours and no lunch breaks among nurses and assistant nurses at an orthopaedic surgery department at a hospital in Sweden, with a particular focus on recovery and psychosocial working environment.

**Methods:**

A qualitative design was used in the study. Eleven nurses and assistant nurses working at the particular orthopaedic department took part in the study, and semi-structured interviews were used to collect data. The interviews were analysed by interpretative phenomenological analysis.

**Results:**

Four main themes were developed in the analysis of the data: A more sustainable working situation, Improved work–life balance, Consequences of being part of a project, and Improved quality of care. Each theme consisted of subthemes.

**Conclusions:**

Overall, reduced work hours appeared to have many, mainly positive, effects for the participants in both work and home life.

## Background

There is a shortage of registered nurses in EU countries, and this shortage is expected to worsen. Job dissatisfaction and ill health are two important factors responsible for the loss of practising nurses [1]. Perceived high work–family conflict has also been identified as a cause of nursing staff turnover, and not surprisingly, long working hours and shift work have been found to be related to work–family conflict among nurses [2, 3]. A large-scale study on work shifts for European nurses found that long work hours had a negative impact on fatigue, health and patient safety [4]. Introduction of reduced work hours is an organisational intervention that could have a positive effect on nurses’ and assistant nurses’ job satisfaction, work–life balance, and willingness to stay in the job.

The six-hour working day and reduced work hours are hot topics that have received increasing attention in the Swedish debate, with supporting arguments focusing on decreasing unemployment and benefits for dual-earner families and non-supporting arguments focusing on reduction of competitiveness of companies and costs for implementation [5]. Despite the considerable interest, there are few studies that have investigated the effects of reduced work hours. A Swedish longitudinal, quasi-experimental study investigated the effects of reduced working hours in a group of social workers [6]. It was found that reduced working hours had a positive effect on several health measures, including restorative sleep, sleep quality (on weekends), stress, memory difficulties, negative emotions, sleepiness, and fatigue and exhaustion (on both workdays and weekends). Moreover, work demands, instrumental managerial support, and work intrusion on private life were also affected positively. Bildt, Åkerstedt and Falkenberg [7] reported the findings from a large-scale study where approximately 400 employees within the public sector in Sweden had reduced work hours (30 h a week). The control group, with approximately 400 individuals, worked 38–39 h a week. Results showed that having reduced work hours was greatly appreciated by the participants, who experienced improved subjective health and quality of sleep and reduced stress and tiredness. However, the study did not find any changes in sick leave or in the biological health markers. A smaller Swedish study investigated a nine-hour reduction of the working week (to a six-hour day) with a comparison group that continued working normal working hours. The participants were mainly female health care and day care nursery personnel. It was found that the group with shortened work hours had improved scores on social factors (time for family, friends, and social activities), sleep quality, mental fatigue, heart/respiratory complaints, and attitude to work hours, whereas the control group did not show changes during the period of the study [8]. A more recent study investigated shortened work hours and exercise during work hours and the effect on productivity for dental staff in Sweden [9]. One group received 2.5 h’ work reduction a week, and the other group received 2.5 h’ work reduction with mandatory physical exercise during the reduced time. Both groups were compared to a control group that did not receive any reduction in work hours or physical exercise. It was found that all three groups increased their productivity, with the largest increase in the group with shortened work hours. Moreover, the group with mandatory physical activity reported the highest self-rated activity. Gothenburg municipality is currently testing a six-hour workday for the staff at a nursing home for the elderly. Another nursing home is included as a comparison group. In the first interim report the preliminary results from the first six months are presented. It was found that the assistant nurses reported more energy and less stress and were more active than before. In addition, the assistant nurses experienced that they were providing better care. A negative aspect was that working many late shifts had a negative effect on sleep [10]. A report that summarised the research on reduced work hours concluded that there is no clear evidence that reduced work hours have any effect on objective health. Subjective health and job satisfaction do, however, appear to be improved [11].

Reducing work hours concurrently with removing breaks could mean a more intense work situation. A well-conducted field study investigated the psychophysiological effects of intensifying work by increasing workload and reducing breaks for driving instructors [12]. The number of examinations during a workday increased from 9 to 11. Having 11 examinations during the day meant a more intense workday and no breaks between the examinations. The physiological activation was measured by adrenaline, and it was found that under the regime of 11 exams a day rates were high after work and stayed so until sleeping time. This was significantly different compared to the situation of 9 exams a day. A conclusion from the study was that the effects of intense work remain after the end of the working day.

A lack of recovery from work plays an important role in stress-related ill health [13]. Recovery can be defined as the time needed to return to normal following the termination of a stressor. Several factors can influence the need for recovery, such as coping factors, health status, private situation, working conditions, and period of time available to recover from work. If there is not enough recovery between work shifts, there may be an accumulation of work-induced fatigue, which increases the risk of ill health [14]. Aronsson, Astvik, and Gustafsson [15] examined the relationship between working conditions, stress, lack of recovery, and health among personnel in the welfare sector. It was found that the individuals in the group ‘not recovered from work’ reported a number of risk factors at work relating to difficult working conditions and reported significantly worse ill health compared to the recovered group. In a literature review on recovery it was found that daily recovery appeared to have a larger effect on health compared to the effects of vacation, where the effects soon disappear. Sleep was suggested to be the most important form of recovery, although it was concluded that more research was needed [16].

Despite the media interest in the topic, there appears to be a lack of research investigating the effects of reducing work hours and eliminating lunch breaks. Indeed, we failed to find any qualitative studies investigating the experiences of having shortened work hours and no lunch breaks for nurses or assistant nurses. Therefore, the aim of this study was to investigate the experiences of reduced work hours and no lunch breaks among a group of nurses and assistant nurses, with a particular focus on recovery and psychosocial working environment.

## Method

### Background

The study took place at an orthopaedic surgery department at a large hospital in Sweden. For a number of years, the department had experienced problems with high turnover and difficulty recruiting nurses and assistant nurses. The staff suggested reducing work hours as a solution to these problems, and the measure was first introduced for a small group in November 2014 and for all staff in February 2015. All nurses (orthopaedic and anaesthetic) and assistant nurses reduced their work hours from eight hours a day to six hours a day, except for one day a week when they still worked eight hours, where the two extra hours were intended for administrative work tasks. Salaries were maintained at the same level as for full-time work. The work schedule was changed from two shifts – a morning shift (7:00–16:00) and afternoon/evening shift (12:30–21:30) – to three shifts – morning (7:00–13:00), afternoon (12:30–18:30), and evening shift (15:30–21:30). In addition, the lunch break was eliminated, which meant that time-consuming respite periods during ongoing operations could be avoided. Extra staff were recruited, and more operating theatres opened, and these could be opened during a longer period of the day compared to previously. As a consequence, more operations were performed, and the idea was that these actions would balance the increased cost of personnel.

### Participants

All assistant nurses and nurses (117) at the particular orthopaedic surgery department were invited via a letter to participate in the study via a letter from the researchers. If the answer was affirmative, the individual was invited to participate in an interview about their experience of reduced working hours. Twelve presumptive participants agreed to take part in the study, which meant a response rate of 10%, and eleven subsequently participated in the interviews. Ten participants were women and one was male, three were working as assistant nurses, and eight were working as registered nurses (specialising in orthopedics or anaesthesia). The ages ranged between 28 and 61 years, and the mean age was 45 years.

### Procedure

Semi-structured interviews were used to collect the data, and the interviews took place at the workplace during working hours in November and December 2015. Two of the authors conducted the interviews (HM and GA), which took between 25 and 55 min. The interview schedule was used as a guide and contained open questions on psychosocial work characteristics, recovery from work, work–family conflict, health, economics, and laws and regulations. The questions in the interview schedule were based on previous literature on psychosocial working conditions and work related health issues for nurses and assistant nurses. The interviews were tape recorded and transcribed verbatim. To keep the interviews anonymous, each participant was assigned a letter as means of identification. The lines of the transcript were numbered, so that each quote presented in this article is referenced with a letter (participant) and a line number from the transcript.

### Data analysis

The interviews were transcribed verbatim and analysed using interpretative phenomenological analysis (IPA) in accordance with the guidelines presented by Smith, Jaraman and Osborn [17]. IPA aims to explore in detail how participants make sense of the world. It is a phenomenological approach that aims to capture the participants’ experiences of the phenomenon that is being studied. Therefore, it was suitable for the current study, because the aim was to explore the participants’ experiences of shortened working hours. The approach is interpretative, as the researcher is trying to understand the experience of the participant through a process of interpretative activity.

In a first step the interview data were coded using pen and paper, line by line in the left margin of the transcript. From this first set of codes more abstract patterns or preliminary themes were condensed for each interview. Then a list of themes from all interviews was created, and themes were grouped together, revised, or deleted. At this stage it was important to check that the analysis was in accordance with the data. After this process a final list of themes and subthemes was developed (see Table 1).

**Table 1:** Main themes and subthemes

Main themes	Subtheme
A more sustainable working situation	Energy for work
	Improved recovery
	Improved work climate
	Effects of having no lunch breaks
Improved work–life balance	Energy for life outside work
	Living life, not just surviving
Consequences of being part of a project	Uncertainty over the future Privilege
	Personnel and recruitment
Improved quality of care	Improved work performance
	Effective use of available resources

## Results

The aim of the study was to investigate the experiences of shortened work hours and no lunch breaks in a group of nurses and assistant nurses, with a particular focus on recovery and psychosocial working environment. In the analysis of the data four main themes were developed: A more sustainable working situation, Improved work–life balance, Consequences of being part of a project, and Improved quality of care. Each theme had a number of subthemes (see Table 1).

### A more sustainable working situation

All the participants experienced that the reduced work hours contributed to create a more sustainable working situation. Several participants were now able to work full-time, as they had sufficient energy to cope with this. They also expressed that they were better able to recover from work and that the work climate had improved. However, there were some negative effects of having no lunch breaks.

#### Energy for work

The reduced work hours gave the staff the opportunity to work full-time while retaining their salary and pension benefits, which was experienced as very positive. Several of the participants talked about how they were able to work full-time, something which had not previously been possible because of difficulty coping with the physical work demands.

*So it [the work] is tiresome and it was… well… yes, one of the reasons why they introduced this, because there were many that couldn’t cope with working full-time. And then you pay for it yourself, both with salary and future pensions. So it is a big trap for women in health care. (registered nurse, E: 110–115)*

One participant explained how they had previously had to work part-time, because the work was too physically demanding for full-time hours.

*Yes, I almost feel better now. My husband can sometimes say, ‘I think you go to work every day’. Yes, now I do, do that. Now I am not off one day a week, I do actually go to work every day. But I can cope with it, I don’t feel, ‘No I can’t cope’… So after almost a year I still feel that I can cope. So hopefully I can work like this until I am 65 years old, because my body can cope with it. (registered nurse, D: 638–650)*

Another aspect of coping with work was experiencing that one’s energy lasted the whole working day. Several participants experienced that, compared to previously, they now had the energy to work in a different way during the whole shift, which meant that they felt that they were doing a better job.

*I think that we deal with the things that need to be done in a better way. I think so… All the materials that arrive from the sterile section should be put away in the shelves. We deal with that in a better way than… We didn’t really have the energy before, perhaps you had forty-five minutes left on your shift and, ‘No, I don’t have the energy to do it, because I am totally exhausted!’ But now I do it… because I have the energy to do it. (registered nurse, D:208–212)*

One participant described feeling that it was impossible to be focused during the full six hours, but that it still worked better than previously.

*You don’t have the energy to keep focus for so long, six hours in one go. Regardless… when I worked eight hours then I had a dip around two, three in the afternoon if I finished at four, then there was a dip when I started looking at my watch. And that dip comes now as well, although it comes at twelve and I finish at one o’clock. (registered nurse, C: 419–423)*

Other participants experienced that it was easier to keep going the last hours, since they knew that the shift would end soon.

*The ordinary evening shifts are half past twelve until half past six, and if I work one of those tough shifts, if I stand in one operation for the whole shift, and start at seven the next day, of course, I will not feel well rested in the morning. But just the thought of knowing that when it is one o’clock I have SO much time to recover, so it is cool. It becomes such a mental thing – you manage to do it because it is such a short shift, even if you are not well rested, because it is such a short period of time until you get to rest. (assistant nurse, J:249–259)*

#### Improved recovery

Many expressed that they felt more recovered from work compared to previously. This was a consequence of both shorter workdays and a different work schedule that allowed for more time between shifts. Before the reduced work hours were introduced, it was common to work a morning shift after an evening shift. Several participants described that they now, because of more available afternoon shifts, could work an afternoon shift after having worked the evening shift. This change was seen as a big improvement, because it was now possible to have sufficient time for recovery between shifts, even when working evening shifts.

*It feels like there is more time for recovery, absolutely. (assistant nurse, F: 257)*

*Like it always has been, all these years, by some tradition in health care, that the one that has worked the evening shift shall start early the next day in order to have some kind of continuity. And it does not have to be this way. We have the right to get a reasonable rest between the shifts… Then, if you need to work at 7:00 once in a while because of some personal reason, no one will forbid you, but the possibility is there, to arrange for a long rest between the shifts. (registered nurse, E: 156–166)*

One person who used to work part-time, described how she worked more days a week compared to previously, but because the days were shorter the daily recovery was better, and there was less need for long weekends to rest.

*If you compare the schedule, now I work here almost every day. If I compare, I used to belong to those who preferred to work many days and then have more time off. Like Friday to Sunday or Saturday to Monday. But for my part I feel better doing this, working shorter days, because when I worked many days I was very tired once I had the days off. (registered nurse, G: 169–179)*

Similarly, another participant explained that working five day shifts in a row previously would have made her exhausted, but that it would not feel as exhausting today because of the shorter shifts.

*If I see one, a week in front of me, where I am working from 7:00–16:00 five days in a row, then I know that I would be a zombie on the Friday. But now, a five-day week, even if it would mean starting 7:00 every morning, although it rarely happens that I have a week with only day-shifts, but if it did happen, it would not feel as burdensome, because I know I get to leave early. (assistant nurse, F: 257–270)*

Several participants experienced that their sleep was improved because they had less stress and ache in the body and were able to sleep without needing to set an alarm.

*I don’t have this back pain, either, like I had previously. It means that my sleep is better. (registered nurse, D: 319–320)*

*I sleep in every day [laugh]. I don’t set the alarm. I go to bed, and I sleep eight to nine hours every night, and it is wonderful. So it feels like I am regaining the sleep that I lost when I was working nights, because at that time it was a constant nagging on the sleep. It just happens. So now, I am rested in a way that I have not felt in many years. (registered nurse, E: 135–142)*

The time off between the shifts increased, which meant an increased possibility for recovery outside work. A consequence of this was being able to let go of work-related thoughts.

*If I work Monday and finish at 13:00 and then start, perhaps I don’t start until 15:30 on the Tuesday or 12:30, and it is quite a long time in between where I have time to rest and let go of thoughts about work. (assistant nurse, F: 231–235)*

#### Improved work climate

The workplace climate had improved, according to participants. They also experienced more stability within the group, as there were fewer people leaving or planning to leave the department. This was experienced as a big change compared to previously.

*I have been here for quite a long time, and there has almost always been someone that has resigned, sometimes several people… But now, and since the summer, I don’t know anybody that has left because they have resigned. I have always known that there are people who are leaving, and this is the first time in a very, very long time, I think, when this isn’t the case. There has always been a big turnover rate. A (registered nurse, A:444–459)*

There was a better climate among the colleagues and more joy and laughter at work, and the participants described how they felt more of a group feeling.

*I think we influenced each other as well, that everybody was in such high spirits. A feeling of euphoria in the whole group. (registered nurse, C: 555–558)*

*“Yes, I think there is more laugher, that people are happier. It is a good atmosphere, for most of the time. It is not always paradise, of course; there are many individuals and there are conflicts here as well, of course. But it feels like there is a happier atmosphere. (registered nurse, E: 547–554)*

*If we get to keep this, and become a unified group, that we… that is very pleasing. We had an after-work a while ago and then one of the guys said that we are a great gang now! Now it is cool, now it is stable! And of course, I become happier when I feel that WE, we are a large group of personnel, and we all work, we strive after the same thing. This is fun. (assistant nurse, J: 605–614)*

Some participants described that there was more collaboration within the department following the reduction of work hours, something that improved the quality of the work.

*I am working at a ward, and everybody works six hours there, and you become a tight team that particular day. So I do think we have become better at collaborating, I do actually think so. When we have to be more focused on what we do. (registered nurse, B: 62–65)*

#### Effects of having no lunch breaks

The participants expressed that the value of having a six-hour workday was higher than the loss of the breaks. However, the lack of lunch breaks was definitely a negative effect of the new work situation, particularly because of hunger. There were differences among the participants regarding their ability to deal with having to go longer without eating. Some got used to it, while others found ways of eating during the shifts.

*[I]t is the only thing that can feel a little hard. And I think it was hard in the beginning, because I am one of those hungry persons (laughter). Here you can take a break when it fits with the work. For example, on the prosthesis side we should avoid opening the doors too much. So we have coffee break very early, and then I am very hungry. So this is something that I have got used to… but is it the only thing that I have felt as being a cost. But it is also worth it. I feel that it is worth it. To know that I can go home at 13:00 and eat then. (registered nurse, G: 216–228)*

*And it sounded heavy at first – omigod, is there no lunch? – but it is so worth it. I get a little hungrier just before lunchtime, but on the other hand I get to come home straight after. Instead of sitting there and having 45-minutes’ unpaid break and going back to work when the alternative is so much more appealing. I can take it. (assistant nurse, F: 334–339)*

*We don’t have any break, but we have to be able to eat. And that has also been a problem, because the doctors have thought – have been a bit annoyed because it is six hours, and when we say that we will eat, they say that we should not eat. Such a miss in communication. Of course we have to eat during six hours, but we don’t have a break, and that is something different. (assistant nurse, H: 340–346)*

A negative aspect of removing the lunch breaks was that the social contact at work was reduced. The participants reported that they rarely got the chance to meet colleagues in the same profession. And some described how they missed talking to colleagues during the lunch break.

*Previously, it could be nice to have time to go to the lunch room and sit down and talk to my colleagues. So that is possibly a negative aspect, that this particular time is gone. But it is so worth it. (registered nurse, E: 194–198)*

*I think it is a little bit of a shame, this with the social aspect. Eh… I think that some people stay after they have finished, if they finish at 13:00, and kind of stay on and eat lunch afterwards. So you get a small part of it, but we rarely see each other, anyway; as an operating nurse you are on your own. You are the only operating nurse in the operating theatre all the time, so you rarely meet and get to speak with the other operating nurses. (registered nurse, C: 299–314)*

Working without taking breaks could make the work appear more intense. Some participants described the days as feeling much shorter than expected.

*When you work six hours you got to keep going all the time (snaps their fingers) until you go home…You do something all the time, and I love it! Time flies. And it never happens that I look at my watch to see what time it is…but suddenly we get relieved and it is over. (assistant nurse, J: 447–455)*

*So when we started with this six-hour day in November I experienced that they days went fast. Time just disappeared, and six hours passed very quickly. Despite the fact that we didn’t have any breaks we thought that the work hours passed very quickly. (registered nurse, B: 20–24)*

### Improved work–life balance

The participants expressed that after the reduced work hours were introduced they had a better balance between work and leisure time, which meant more time and energy for leisure activities and for non-work-related everyday tasks. Moreover, the stress of everyday life was easier to handle, and some experienced an increased level of control in their lives, as they had more control over what shift to choose. Finally, a few participants expressed that they now lived a more ideal life compared to previously.

#### Energy for life outside work

Many expressed that there was more energy for life outside of work. Some expressed that work used to consume all their energy and that there used to be no energy left after work, whereas now there was energy for both work and leisure time.

*So I cannot see any disadvantage of working six hours. I can only see advantages with it. I can work full-time, I don’t wear down my body, I get a good balance. I think that as a human you only have the energy to work a certain part of the day… Previously, all the energy went to work and there was nothing left. Now there is a better balance, I have the right amount of tiredness when I go to work. (registered nurse, D: 480–492)*

Several participants described having more time and energy to spend time with family and friends.

*I can say that I have more energy and desire to meet people during the weekends when I am off. Previously (before reduced work hours), I could feel that I didn’t have the energy, you know. (registered nurse, I: 178–182)*

*I do have the energy to call friends and spend time with them. I still have the desire to do things – as you do when you are not too tired. (registered nurse; K, 148–151)*

Shorter working hours meant more time and energy, which some of the participants spent doing physical exercise.

*I am not physically tired, because I work six hours. I still have energy in my body and can go to the gym afterwards. (registered nurse, D: 85–86)*

*We work such short days, so we have time to do many other things as well, I have time to do a lot of other physical activity. I think that … my body is stronger now, after a year, compared to when we started with this. So… I have time to take care of my body. So I don’t have massive pain in my shoulders and lower back anymore. (registered nurse, B: 171–178).*

Some used the extra spare time to relax on the beach.

*[I]f I start at 15:30, then I cycle out to the sea and lay there until 14:00, and then cycle to work. I get – it feels like I have two days in one. I have the time to have a whole lot of spare time and a whole lot of work. (assistant nurse, J: 120–125)*

Spending less time at work meant having more time to do other things that needed to be done during the day, such as dentist appointments and domestic chores. To have the morning or the afternoon to deal with these things appeared to create more flexibility, which made everyday life easier and less stressful.

*…I think I have a good balance. It feels like everyone else says that they just work, that life is all about work, but I don’t feel that way, anymore. Or, it is like this: I have two parts, I have work and leisure time every day… It is very nice actually. Today I will have time to go to the shops, prepare for Christmas, and clean, and then I will still have my two hours on the couch. (assistant nurse, J: 621–630)*

*I have the time, and therefore I can plan these things, talks with teachers, visits to the dentist. Normally, I need to take time off… but if I start at noon, then I have four hours before lunch… Even if I start at twelve I am home at 19:00. So I am still at home in time to eat dinner with my family and help at bedtime. So from a family point of view this is great. (registered nurse, C: 71–79)*

Some participants related that they had previously experienced domestic stress relating to everything that needed to be done outside of work, and that this stress now was gone.

*I feel that there is less stress… It is only positive, there is nothing negative at all… I think that is the greatest gain. Because to come home after four o clock and start cooking food, and then there is homework and stuff, suddenly the time is way too much, and I should have managed to do a little more… Time just flies. And it is not like that now. (registered nurse, I: 509–521)*

Another aspect of the reduced work hours was that the participants who had previously worked part-time, in order to manage everyday life, were now able to work full-time. This had a positive effect on their pay and pension benefits.

*If I had worked here and it was eight hour shifts, I would have worked part-time. Then I would have worked 80% – I would not have managed to work 100%, eight hours – I would not have managed that. Because then my daughter would be at nursery for a very long time and it would have been… difficult. It is tiring I have worked 100% as a nurse before I had my child, at that was ok. But since then I have worked part-time… Now I can work full-time with all that it entails regarding pension and salary and so on, but still have the advantages of reduced work hours. (registered nurse, F: 64–74)*

#### Living life, not just surviving

All participants expressed that their life situation had improved and that there was more time to do what they really wanted. They appeared to be living life in a manner that was closer to an ideal way of living.

*I feel that it must be a little bit more like this that one is supposed to live. Instead of working all day, going home and picking up tired children and what not. No, I, because I feel that I have another form of energy, I come home and it is afternoon and I have time to be in the sun and take care of things. And socialise, and spend time with the family. (registered nurse, G: 602–612)*

*You get a life. I can say as much. You do get it, actually – it is unbelievable what a big difference these two hours make. (registered nurse, B: 460–465)*

A consequence of gaining a better balance was the experience of living a fuller life.

*Everything is about work, and it is your life… Work is a big part, but you still have to have the energy to live! That is the primary thing, and if you have the energy to live and feel that you have the energy to manage things, then you are happy. And, of course, when you feel good, that will have an influence on work. (registered nurse, K: 61–167)*

### Consequences of being part of a project

The reduction of work hours within this department is a time-limited project that was planned to run for two years. It will be evaluated and possibly become a permanent change. Because of this situation, the participants described uncertainty over the future of their employment and the department. However, their descriptions of the project were full of enthusiasm for reduced work hours, and it was seen as a privilege that they were willing to make sacrifices for.

#### Uncertainty over the future

Because the project is time limited, many participants expressed that there was a feeling of uncertainty among the staff. Many were worried about what would happen in the future and predicted a high risk of staff leaving the workplace if the reduced work hours were to be discontinued.

*If they stop this and decide that we will not continue to get it (reduced work hours), anymore, then people will flee this place… Because it is orthopaedic surgery we are working with and many think it is demanding work… so these six hours make up for that. (registered nurse, D: 687–693)*

*I think things could turn out really bad for this workplace if that were to happen (that reduced work hours would be discontinued)… Because, as I have said, this (reduced work hours) has raised the place so much… I am not sure, but I think that many would quit. (registered nurse, G: 322–327)*

The main determinant of whether the project would be continued was the cost-effectiveness of the programme, according to the participants. Cost-effectiveness in this context meant producing more care to compensate for the increased costs linked to reduced work hours. The participants believed that the well-being of the personnel was not enough to compensate for the increased costs.

*It is not enough that we, the personnel, are satisfied, it is not enough. Instead, it has to be shown, that we have a high production, that it is worth it, to take care of all people, so nobody should have to wait, and nobody should have to be sent to other hospitals, which costs money… That we can be cost-effective. And that through this we can see that this is worth it. (registered nurse, K: 43–49)*

*We have produced a lot more, but it is still money in the end, and, yes, they have to do their estimations, and so, I don’t know, nothing is certain. (registered nurse, G: 316–320)*

The belief that the economy was the most important factor appeared to create an expectation of increased effectiveness and productivity, something that could lead to feelings of stress.

*[T]his is a project, and we are not sure that we get to keep it. So I am on my toes, for sure, I don’t want to laze around. I wouldn’t do that normally, either, but do I hurry up. (assistant nurse, J: 414–419)*

*Now, there is a goal that we should become more effective, we shall work, do more, because everyone wants to keep the six hours… so then I do shape up (assistant nurse, H: 134–137)*

*I know some that think that the pace is much higher, that they feel more stress. That is not my experience… Organisational change can be stressful, but here it has been so positive. (assistant nurse, F: 535–538)*

#### Privilege

A pervading theme in the participants’ accounts was the expression of elation and enthusiasm for the reduced work hours project. They described a feeling of being chosen and having been given a privilege that they wanted to make the best of.

*I think we have a great situation… We say that several times to each other, God, we got it so good! Now we are going home at 1 p.m. Great! (assistant nurse, F: 35–38)*

*Because it is still a sign of trust, a gift that we have received, a possibility. That one wants to make the best of. (registered nurse, K: 356–357)*

Some of the participants stated that the new work hours were such a positive change that it would be difficult to consider working anywhere else if the shortened work hours became permanent.

*A negative aspect was that – haa, now I can’t work anywhere else, if I should ever have those thoughts, because this is too damn good… I guess I get to stay here (laughs), because it is difficult to give this up, as it is fantastic. (registered nurse, E: 533–539)*

*I feel a little worried about whether I will ever dare to leave this place if we get to keep the six hours… Will I hold on to this and not dare (laugh), dare to move and try something else? (registered nurse, I: 589–593)*

A common theme in the interviews was that the reduced work hours had meant so many positive things in the participants’ lives that they preferred to endure the negative aspects and problems linked to the reduced hours rather than going back to normal working hours.

*To go back and work nine hours … with a lunch, it is not an option for me, if I get to choose this. Despite nights and a non-existent management. Despite a lot of different things, such as severely ill patients and other things. (registered nurse, C: 334–337)*

*Personally, I think it is fantastic to work six hours. It is intense and you don’t get a break, but that does not bother me at all. So I think that in life this suits me really well. (assistant nurse, H: 36–41)*

*There are a lot of great things with this… Some people you don’t see for weeks, sometimes. But still I have to say that I would not swap this for anything in the world. I really hope it will be continued. (registered nurse, B: 677–685)*

Many of the participants experienced the reduced work hours as something very positive, and they believed that more people should have the opportunity to try it.

*The more you look at it, the more you realise that this is something that everybody should have… I think… I do actually think that everybody experiences positive effects from it. (registered nurse, D: 784–789)*

*I hope that it will be continued and that there will be more that dare to try it. (registered nurse, G: 595)*

#### Personnel and recruitment

The participants described how the reduced work hours had involved hiring more staff to fill out the new shift schedules. Previously, the department had had problems recruiting and had needed to hire temporary staff from staffing agencies. Several of the participants reported that the reduced work hours were a big reason they started working at the department. Another aspect that was highlighted in the interviews was the possibility of attracting new staff through reduced work hours rather than with salaries.

*It was actually this project that attracted me, to have the opportunity to be part of it, to work six hours and try that… So that was the crucial … that I applied here. Mm, so I was here from the beginning when it started. (registered nurse, K: 13–18).*

*To compete with the salary is not done that much… rather it is, oh, how can we get the staff? And it is so difficult within certain areas within health care. It really is. And this is a fantastic way of recruiting personnel. It is a big gain. (registered nurse, K: 567–573)*

*Previously, they have not been able to recruit people. They have had many, especially operating nurses – there have been so many temporary nurses from staffing agencies. And it must have been expensive having them all. Now suddenly… now they start to work, recently graduated, though, but they are really great, those girls and boys. They started recently five or six, and this attracts people, it really does. (registered nurse, D: 677–685)*

The participants described how the newly recruited staff have had a positive effect on the atmosphere in the department.

*And it is people that have been attracted to this place because of this… We, the people that have started are bringing in an amazing energy and positive atmosphere… that I believe has had a positive effect on the whole department. And it is not only the case that they can attract people – they can attract very good people. (registered nurse K: 575–582)*

### Improved quality of care

All throughout the interviews the participants described that there was an improvement in the quality of care due to the new shifts that were introduced together with the reduced work hours.

Several factors led to this improvement, including an improved performance of the staff and better use of available resources such as operating theatres.

#### Improved work performance

Some participants reported that they had improved their work performance following the reduced work hours. For example, they described being focused on doing a good job during the operations.

*You have one or two operations. And you got to finish those. And you can do it really great. I do think that I am doing a qualitatively better job now compared to previously. I have always aimed to do a good job, but I think that my work is even better now, actually. I do actually believe this… because I know that ‘This is the operation that I shall do, then do it well.’(registered nurse, B: 277–283)*

Some participants reported that the staff had more energy following the reduced work hours and were therefore able to engage more with the patients compared to previously, when they were more tired.

*I think that people were more exhausted and found it more difficult to motivate themselves to do a good job, compared to now. Now I believe that several feel that they have the energy to engage with the patients and do the job we are supposed to do. (registered nurse, C: 641–643)*

Several of the participants said that the new shifts have meant fewer door openings during the operations, which decreases the risk for infection. In addition, a further benefit of having fewer changes of staff during the operations was the reduced risk of misunderstandings and mistakes.

*The fewer changes you have, the fewer times the door is open, the fewer people that are involved, the less risk of misunderstandings or something being forgotten or misunderstandings. In fact, the more people that are involved in something, the more potential mistakes. (registered nurse, B: 116–121)*

#### Effective use of available resources

The new shifts allowed the department to use the operating theatres during a larger part of the day, according to the participants. Previously, the work had to be wrapped up early in the afternoon, because the staff were finishing work in the afternoon. The participants proudly described how the department had increased its activity and how the rooms were now better used.

*[T]hen we had to… start wrapping up early at several operating theatres so that we were done by three o’clock, because people finished work at four o’clock. And then you can’t start the next operation, because it would take too long. So the operating theatres were not effectively utilised, and that is also a big cost… having them stand empty cost a lot. (registered nurse, E: 320–330)*

*[T]he six-hour workday and the increased number of staff has meant that we can open another operating theatre… which means that our activity has increased. (registered nurse, E: 390–392)*

In addition, the participants experienced that the time they spent at work was used more efficiently compared to previously. The time they called ‘wasted’ had been reduced, and the elimination of breaks meant that they no longer needed to replace each other during lunch breaks, something that used to take a lot of time.

*I was going to replace her (during a surgical procedure)… And then I had to get into the work and all the equipment that is needed for the operation, and then she comes back forty-five minutes later… and then I would go out again and report to her what has happened during the hour. No it was not good. This way is much better. Now I replace her and she goes home, and I stay until it is finished. (registered nurse, D: 246–257)*

*In my experience we are more effective than when we worked eight hours. Because it was a lot of looking at the watches: ‘Oh, soon there is lunch,’ a little bit like that. (registered nurse, B: 74–77)*

## Discussion

The current qualitative study investigated the experiences of reduced work hours and no lunch breaks among nurses and assistant nurses at an orthopaedic surgery department in Sweden. Four main themes with a number of subthemes were found in the interview data. The main themes were A more sustainable working situation, Improved work–life balance, Consequences of being part of a project, and Improved quality of care. All participants in the study appeared to view the reduced work hours as a positive change at work.

‘A more sustainable working situation’ consisted of a number of subthemes, including ‘Improved recovery’. According to the participants, working shorter days improved recovery in several ways. Work consumed less energy because the participants spent less time at work, and there was more time for recovery before and after the work shifts. Thus, the workdays were shorter and the periods outside work were longer, which created good opportunities for continuous recovery from work. Indeed, previous research has found that daily recovery is more important for health than longer vacations. The positive effects of a vacation quickly disappear [16]. However, in the present study the lunch breaks were eliminated, and shorter breaks/pauses were not guaranteed. In the subtheme ‘Effects of having no lunch breaks’ the participants reported that the reduced work hours compensated for the lack of breaks. The lack of breaks was not described as a big problem for the staff. A reason for this could be that the staff had been involved in the planning of the new work hours and shifts, which meant that they had a certain level of control over the work situation. The lack of breaks most likely involved intensifying work, which can have negative health effects. In a study with driving instructors it was found that removing shorter breaks and intensifying the work had negative effects on sleep and cognitive performance [12]. However, the work hours were not reduced in that study. In addition, a study with social workers found that reduced work hours had a positive effect on restorative sleep, sleep quality (on weekends), stress, sleepiness, and fatigue and exhaustion (both on workdays and weekends). Moreover, instrumental manager support was also affected positively, which is somewhat in accordance with the present study, which found an improved work climate [6].

In the main theme ‘Improved work–life balance’ the participants described an improved balance between work and leisure time. Improved balance between work and life involved several aspects, such as more energy to do things during leisure, time including increased social activity and exercise. Indeed, a previous study on the effects of the six-hour workday, for female health care and day care personnel, found that the biggest effect was increased time for social activity [8]. However, the same study did not find an increase in exercise. The participants in the current study also reported that it was easier to handle the everyday stress with children and family commitments following the reduced work hours and that they experienced an increased control over working hours. The previously mentioned study found that reduced work hours had a positive effect on work intrusion on private life [6]. Previous research has shown that a poor work–life balance, regardless of gender, is related to having more health problems compared to employees with a good work–life balance [18].

‘Consequences of being part of a project’ was a main theme related to the fact that the reduced work hours project was introduced on a temporary basis with no definite end date. The participants reported experiencing uncertainty and worry over what would happen in the future expressed a strong wish for the project to continue. The uncertainty and lack of control regarding what would happen with the working situation in the future appeared to lead to worry among the participants. Indeed, control is an important factor in well-being at work, and lack of control can have negative effects on health [19].

The final main theme was ‘Improved quality of care’, which described how the participants reported that they had improved their performance and that the department’s resources were used more effectively following the reduced work hours. Indeed, increased production and employee health do not need to be the only measures of the effects of reduced work hours. Quality of care could also be a relevant outcome measure. The preliminary results in the evaluation of a six-hour workday at a nursing home for the elderly in Gothenburg municipality found that the assistant nurses experienced that they were providing better care [10]. Another study that evaluated reduced work hours compared to physical activity at work found that the group that had reduced work hours had improved their productivity more than the group that did physical activity [9].

There are several limitations with this study, one being the low response rate, with only 10% of the members of staff agreeing to take part in the study. It could be the case that the participants who agreed to take part were the members of staff that were particularly positive or negative towards the project. It is also possible that the participants wished to portray the project in a good light during the interviews for fear of a negative evaluation of the project. These risks need to be considered, but the interviewers were very aware of these possibilities and asked about both positive and negative aspects of the participants’ experiences. In, addition a structured interview questionnaire could have assisted the data analysis.

Transferability is important to consider in qualitative studies. Issues such as the sample and number of groups and interviews need to be considered. The sample consisted of individuals who worked as nurses or assistant nurses with reduced work hours at a particular department. Only a small proportion of the staff at the department took part in the study, so it is not possible to say that the findings represent all individuals. However, this is in accordance with the method of IPA, where studies are conducted with small sample sizes, and through purposive sampling a group of participants is found for whom the research question is significant [17]. Regarding generalisability, it could be said that if the study has identified an experience, it could be similar for many others. For the current study it is also important to relate the finding to previous studies (see above) and thereby add to the accumulation of results regarding the experience and effects of reduced work hours [20].

Possible implications of these findings are that the reduced work hours could help to create a more sustainable working situation for nurses and assistant nurses. There may be positive long-term effects on both physical and psychological health that is not yet evident. Reduced work hours increase the possibility for sufficient recovery between the shifts. However, some participants found it difficult to work a whole shift without a break (although they still thought it was worth it), and for future studies it could be important to consider the issue of recovery or lack of recovery while working for six hours without a break. Perhaps a formal system for food intake during the shifts could be useful. There have been difficulties recruiting nurses within this particular region, and another implication could be that implementing reduced work hours is a way to create an attractive place of work for nurses that would make the recruitment process easier. This particular hospital could not compete for staff with higher salaries than other departments, so offering reduced working hours could be one effective way to attract skilled personnel. Furthermore, the participants expressed that they were now able to provide better care for their patients, and this could also be a possible effect of reduced work hours.

Implications for nursing practice research is that future studies should evaluate short- and long-term individual and organisational effects of reduced work hours including quality of care. Considering this is a qualitative study, that cannot be generalised to the same extent as larger quantitative studies, further studies are needed to draw firm conclusions regarding the effects of reduced work hours and to make suggestions for nursing practice research.

## Conclusions

The present study explored the experiences of a number of nurses and assistant nurses who had reduced work hours. Four main themes were found: A more sustainable working situation, Improved work–life balance, Consequences of being part of a project, and Improved quality of care. Overall, reduced work hours appeared to have many, mainly positive, effects, in both work and home life.
